# Kir6.1/K-ATP channel modulates microglia phenotypes: implication in Parkinson’s disease

**DOI:** 10.1038/s41419-018-0437-9

**Published:** 2018-03-14

**Authors:** Ren-Hong Du, Hong-Bin Sun, Zhao-Li Hu, Ming Lu, Jian-Hua Ding, Gang Hu

**Affiliations:** 1 Jiangsu Key Laboratory of Neurogeneration, Department of Pharmacology, Nanjing Medical University, 101 Nongmian Avenue, Nanjing, 211166 P.R. China; 2 Department of Pharmacology, Nanjing University of Chinese Medicine, 138 Xianlin Avenue, Nanjing, 210023 P.R. China

## Abstract

Classical activation (M1 phenotype) and alternative activation (M2 phenotype) are the two polars of microglial activation states that can produce either neurotoxic or neuroprotective effects in the immune pathogenesis of Parkinson’s disease (PD). Exploiting the beneficial properties of microglia cells by modulating their polarization states provides great potential for the treatment of PD. However, the mechanism that regulates microglia polarization remains elusive. Here we demonstrated that Kir6.1-containing ATP-sensitive potassium (Kir6.1/K-ATP) channel switched microglia from the detrimental M1 phenotype toward the beneficial M2 phenotype. Kir6.1 knockdown inhibited M2 polarization and simultaneously exaggerated M1 microglial inflammatory responses, while Kir6.1 overexpression promoted M2 polarization and synchronously alleviated the toxic phase of M1 microglia polarization. Furthermore, we observed that the Kir6.1 deficiency dramatically exacerbated dopaminergic neuron death companied by microglia activation in mouse model of PD. Mechanistically, Kir6.1 deficiency enhanced the activation of p38 MAPK–NF-κB pathway and increased the ratio of M1/M2 markers in the substantia nigra compacta of mouse model of PD. Suppression of p38 MAPK in vivo partially rescued the deleterious effects of Kir6.1 ablation on microglia phenotype and dopaminergic neuron death. Collectively, our findings reveal that Kir6.1/K-ATP channel modulates microglia phenotypes transition via inhibition of p38 MAPK–NF-κB signaling pathway and Kir6.1/K-ATP channel may be a promising therapeutic target for PD.

## Introduction

Parkinson’s disease (PD), the second most common neurodegenerative disorder after Alzheimer’s disease, is characterized by the progressive loss of dopaminergic (DA) neurons in substantia nigra compacta (SNc) and excessive reactive microgliosis^1^. Overwhelmingly activated microglia are observed in the vicinity of the degenerating neurons in the SNc of animal models as well as in PD patients^2,3^. Microglia-mediated neuroinflammation is an important component in PD pathogenesis. However, simple anti-inflammatory strategy may not be efficacious in clinical therapy of PD.

Microglia activation can be classified into two major phenotypes defined as ‘classical activation’ (also termed M1 phenotype) and ‘alternative activation’ (M2 phenotype)^4–6^. M1 microglia polarization is associated with the production and release of multiple pro-inflammatory cytokines^7,8^. The released factors generally act in tissue defense and promote the destruction of pathogens^9^. However, overactivated or dysregulated microglia are constantly involved in the pathogenesis of PD and serve to amplify neuronal damage caused by pathological stimuli and toxins, which in turn, induces more widespread damage to the neighboring neurons^10^. In contrast to the M1 phenotype, M2 microglia executes an anti-inflammatory effect and promote wound healing and tissue repair. The major anti-inflammatory cytokines, such as interleukin-4 (IL-4), IL-13, IL-10 and transforming growth factor-β (TGF-β), initiate the alleviation of pro-inflammatory responses and enhance the expression of genes that are involved in tissue recovery and repair. This resolution state is critical in chronic neuroinflammation-related diseases including PD^11–13^. As the two microglia phenotypes can transit each other in different pathogenetic stages of PD, it might be available to make microglia protective by switching their phenotypes^14,15^. Therefore, for the future treatment of PD, it will be an effective strategy to halt the toxic phase of M1 microglia polarization and restore tissue homeostasis by switching the microglia phenotypes or enhancing the beneficial effects of M2 microglia^16^.

ATP-sensitive potassium (K-ATP) channels, the unique channels coupling cell metabolism to cell membrane potential, are hetero-octamers composed of pore-forming Kir6.x (6.1 or 6.2) subunits and sulfonylurea receptor (SUR1 or SUR2) regulatory subunits, regulated by intracellular ATP and ADP concentrations^17^. As a metabolic sensor, K-ATP channels are widely expressed in most metabolically active tissues, including brain^18^, heart^19^ and pancreatic β-cells^20^. Within the brain, Kir6.2 is predominantly expressed in neurons^21^ and Kir6.2 knockout resulted in a rescue of SNc DA degeneration in mouse models of PD model^22^. Kir6.1 is mainly expressed in microglia and astrocyte^23,24^. Our previous study showed that Kir6.1/SUR2 K-ATP channels were expressed in microglia and opening of microglial K-ATP channels could alleviate rotenone-induced degeneration of DA neurons via inhibition of neuroinflammation^25^. However, the contribution of Kir6.1-containing K-ATP (Kir6.1/K-ATP) channel to microglia phenotype and PD remains unknown. In the present study, we demonstrate that Kir6.1/K-ATP channel is essential for M2 microglia polarization. Kir6.1 deficiency switches microglia from the beneficial M2 phenotype into the detrimental M1 phenotype, which finally accelerates DA neuron death in mouse models of PD model.

## Results

### Kir6.1 deficiency aggravates the loss of DA neuron via promoting excessive microglia overactivation in SNc of 1-methyl-4-phenyl-1, 2, 3, 6-tetrahydropyridine (MPTP) PD model mice

**Fig. 1:**
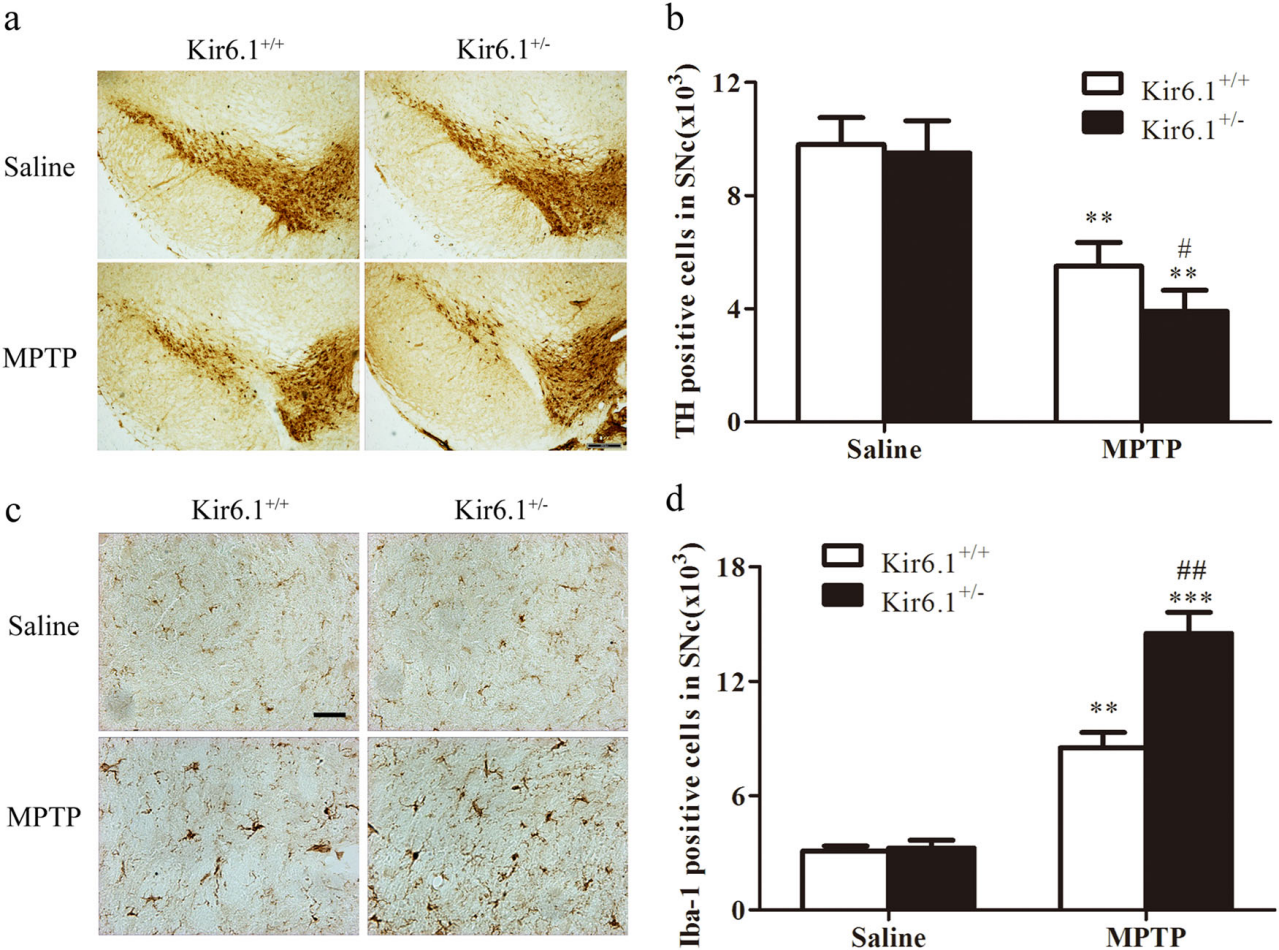
Kir6.1 deletion aggravated dopaminergic neuron loss and microglia overactivation in MPTP Parkinson’s disease model mice. **a** Microphotographs of Tyrosine hydroxylase (TH)-positive neurons in the substantia nigra compacta (SNc). **b** Stereological counts of TH-positive neurons in the SNc. **c** Microphotographs of ionized calcium-binding adaptor molecule 1 (IBA-1)-positive cells in the SNc. **d** Stereological counts of IBA-1-positive cells in the SNc. Data are presented as mean ± SEM, **p < 0.01, ***p < 0.001 versus corresponding control (saline) group; ^#^*p* < 0.05, ^##^*p* < 0.01 versus MPTP-treated Kir6.1^+/+^ groups. *n* = 6 for each group. MPTP 1-methyl-4-phenyl-1, 2, 3, 6-tetrahydropyridine

Since Kir6.1 homorozygotes mice can not completely endure the stress of PD models, Kir6.1 heterozygotes mice were subjected to the MPTP PD models to study the role of Kir6.1 in PD and the member of SNc DA neurons was counted. Stereological counts of SNc DA neurons defined by Tyrosine hydroxylase (TH) staining showed no difference in SNc TH cell number between mice of both genotypes under saline treatment. MPTP treatment decreased TH-positive cells by 43% in SNc of Kir6.1^+/+^ mice, but decreased TH-positive cells by 58% in Kir6.1^+/−^ mice (Fig. 1a, b). These data indicate that Kir6.1^+/^^−^ mice are significantly more prone to MPTP-induced neurotoxicity than their Kir6.1^+/+^ littermates.

As microglia-mediated neuroinflammation plays crucial roles in DA neuron death, we investigated microglia activation by using immunofluorescence to detect their marker, ionized calcium-binding adaptor molecule 1 (IBA-1). As shown in Fig. 1c,d, Although MPTP injection led to extensive microglia overactivation in both genotypic mice, the population of overactivated microglia with ameboid morphology was larger in Kir6.1^+/−^ mice (5-fold elevation) than that in Kir6.1^+/+^ mice (3-fold elevation). Taken together, these results suggest that Kir6.1 deficiency accelerates DA neuron degeneration in the MPTP mouse model by facilitating excessive microglia overactivation.

### Kir6.1 deletion facilitates the switch of microglia phenotypes from M2 to M1 in SNc of MPTP PD model mice

**Fig. 2:**
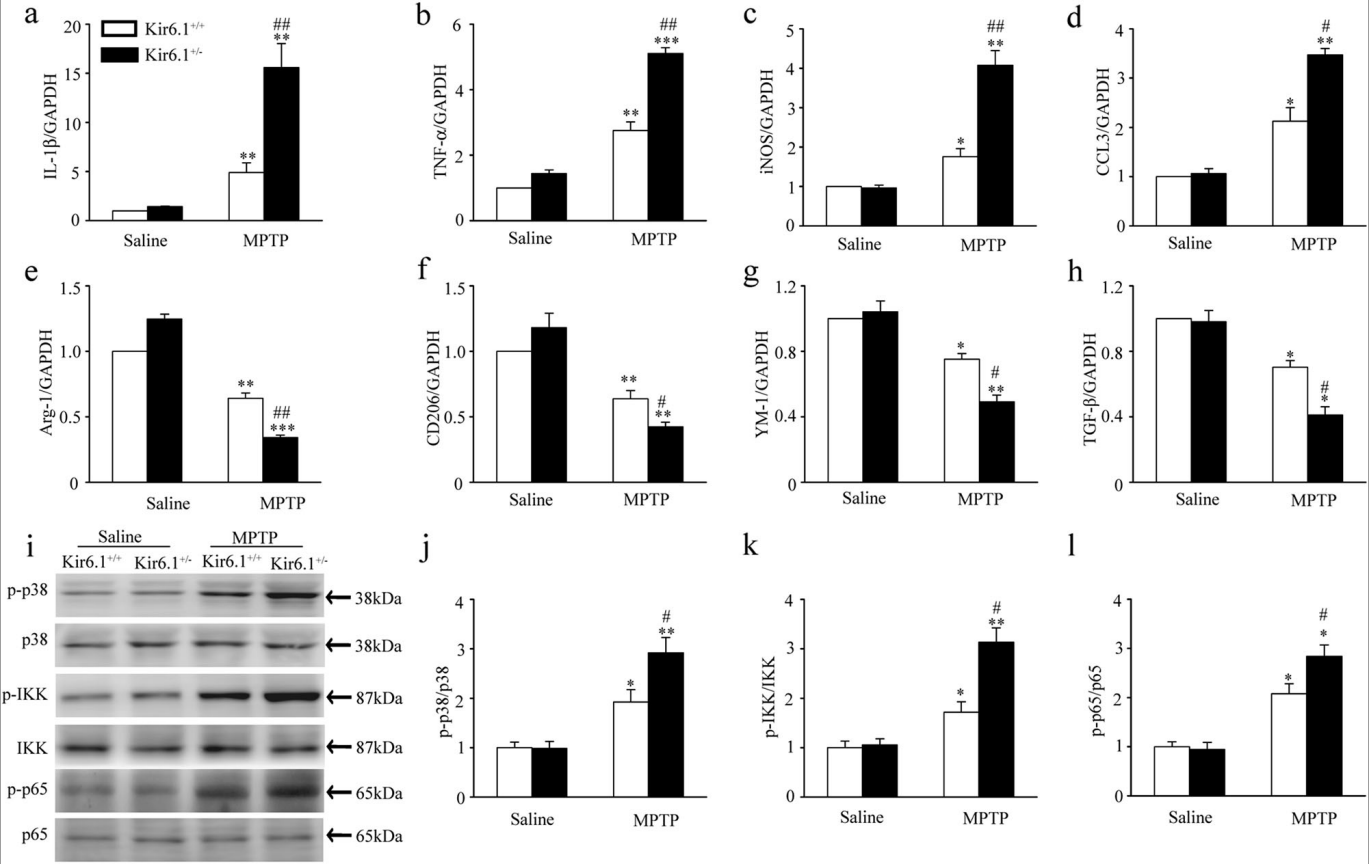
Kir6.1 deficiency facilitated the switch of microglia phenotypes from M2 to M1 in MPTP Parkinson’s disease model mice. **a**–**h** Kir6.1 deficiency increased the production of IL-1β (**a**), TNF−α (**b**), iNOS (**c**), and CCL3 (**d**), and reduced the expression of Arginase1 (**e**), CD206 (**f**), YM-1 (**g**) and TGF-β (**h**) in the substantia nigra compacta (SNc). **i**–**l** Kir6.1 deficiency enhanced the activation of p38 and NF-κB in the SNc. Representative immunoblot (**i**) and quantitative analysis of the phosphorylation of p38 (**j**), IKK (**k**) and p65 (**l**) in the SNc of Kir6.1^+/+^ and Kir6.1^+/^^−^ mice. Data are presented as mean ± SEM, *p < 0.05, **p < 0.01, ***p < 0.001 versus corresponding control (saline) group; ^#^*p* < 0.05, ^##^*p* < 0.01 versus MPTP-treated Kir6.1^+/+^ groups. *n* = 5 for each group. MPTP 1-methyl-4-phenyl-1, 2, 3, 6-tetrahydropyridine

Microglia phenotypes can be distinguished by their expression of characteristic surface marker genes^26^. By comparing the mRNA levels of those markers and related molecules in both genotypic mice, we showed that the M1 markers such as IL-1β, TNF-α, iNOS, and CCL3 were significantly increased in the SNc of Kir6.1^+/^^−^ mice compared to Kir6.1^+/+^ mice following MPTP challenge (Fig. 2a–d), whereas the M2 markers including Arginase1, CD206, YM-1 and TGF-β were generally decreased (Fig. 2e–h). These data demonstrate that Kir6.1 deletion promotes the switch of microglia phenotypes from M2 to M1 in the pathogenesis of PD. As MAPK and NF-κB play important roles in microglia-mediated neuroinflammation^27^, we then detected the expression of MAPK and NF-κB pathway in the SNc of both genotypic mice. It was found that Kir6.1^+/-^ mice displayed a marked increase in the phosphorylation of p38, IKK and p65 but not the total of p38, IKK and p65 compared with Kir6.1^+/+^ mice after exposure to MPTP (Fig. 2i–l). The results suggest that Kir6.1 deficiency switches microglia phenotypes from M2 to M1 via activation of p38 MAPK–NF-κB signaling pathway.

### Kir6.1 enhances M2 microglia polarization

**Fig. 3:**
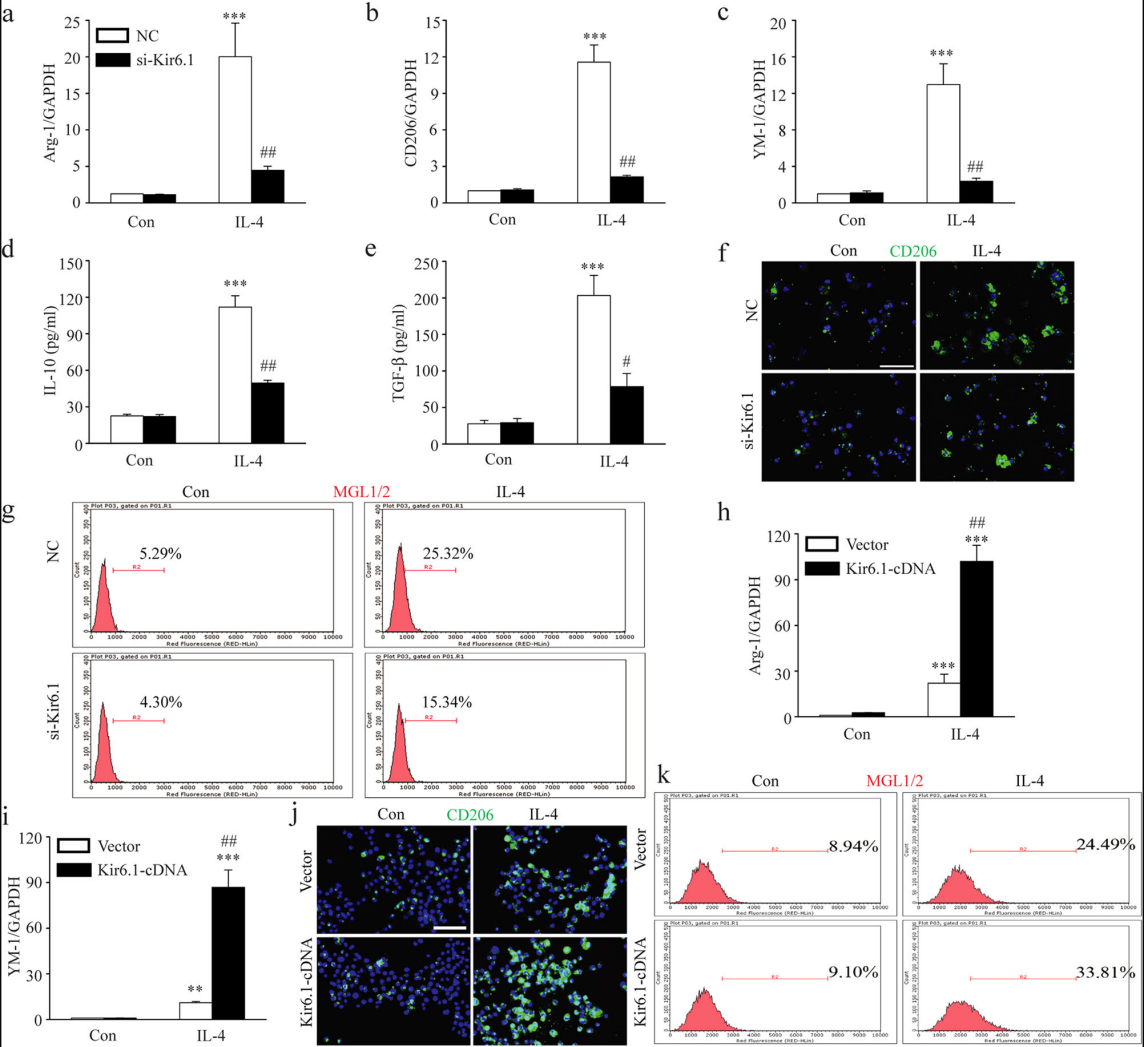
Kir6.1 promoted M2 microglia polarization. **a**–**g** Kir6.1 knockdown reduced the expression of related M2 markers. The expression of Arginase1 (**a**), CD206 (**b**), YM-1 (**c**), IL-10 (**d**) and TGF-β (**e**) were assessed by qPCR or ELISA in microglia. **f** Representative immunofluorescence staining of CD206 was visualized under microscopy. **g** The expression of M2 related biomarker MGL1/2 was detected by flow cytometry. Data are presented as mean ± SEM from four independent experiments, ***p < 0.001 versus corresponding control; ^#^*p* < 0.05, ^##^*p* < 0.01 versus IL-4-treated negative control (NC) groups. **h**–**k** Kir6.1 overexpression increased the expression of related M2 markers. The expression of Arginase1 (**h**) and YM-1 (**i**) were assessed by qPCR. **j** Representative immunofluorescence staining of CD206 was visualized under microscopy. **k** The expression of M2 related biomarker MGL1/2 was detected by flow cytometry. Data are presented as mean ± SEM from four independent experiments, **p < 0.01, ***p < 0.001 versus corresponding control; ^##^*p* < 0.01 versus IL-4-treated vector groups

To explore the roles of Kir6.1 in M2 microglia polarization, we employed both loss-of-function and gain-of-function strategies. BV2 microglia was stimulated with IL-4 and the M2 gene expression was detected using real-time PCR and ELISA. As shown in Fig. 3a–e, the increased expression of M2 markers including Arginase1, CD206, YM1, TGF-β and IL-10 initiated by IL-4 treatment was significantly repressed in the Kir6.1 knockdown cells. Consistently, the expression of CD206 was greatly reduced in the knockdown cells after IL-4 treatment determined by immunofluorescence analysis (Fig. 3f). In addition, flow cytometry analysis also showed that M2 specialized gene MGL1/2 was significantly attenuated in Kir6.1 knockdown microglia treated with IL-4 (Fig. 3g). On the contrary, the levels of both Arginase1 and YM1 were greatly increased when Kir6.1 was transiently expressed following the IL-4 challenge (Fig. 3h, i). In line with the real-time PCR result, the expression of CD206 was accordingly elevated upon Kir6.1 overexpression (Fig. 3j). Moreover, Increased Kir6.1 expression dramatically resulted in a significant increase in the expression of MGL1/2 induced by IL-4 (Fig. 3k). These results show that Kir6.1 promotes M2 microglia polarization.

### Kir6.1 inhibits M1 microglia polarization

**Fig. 4:**
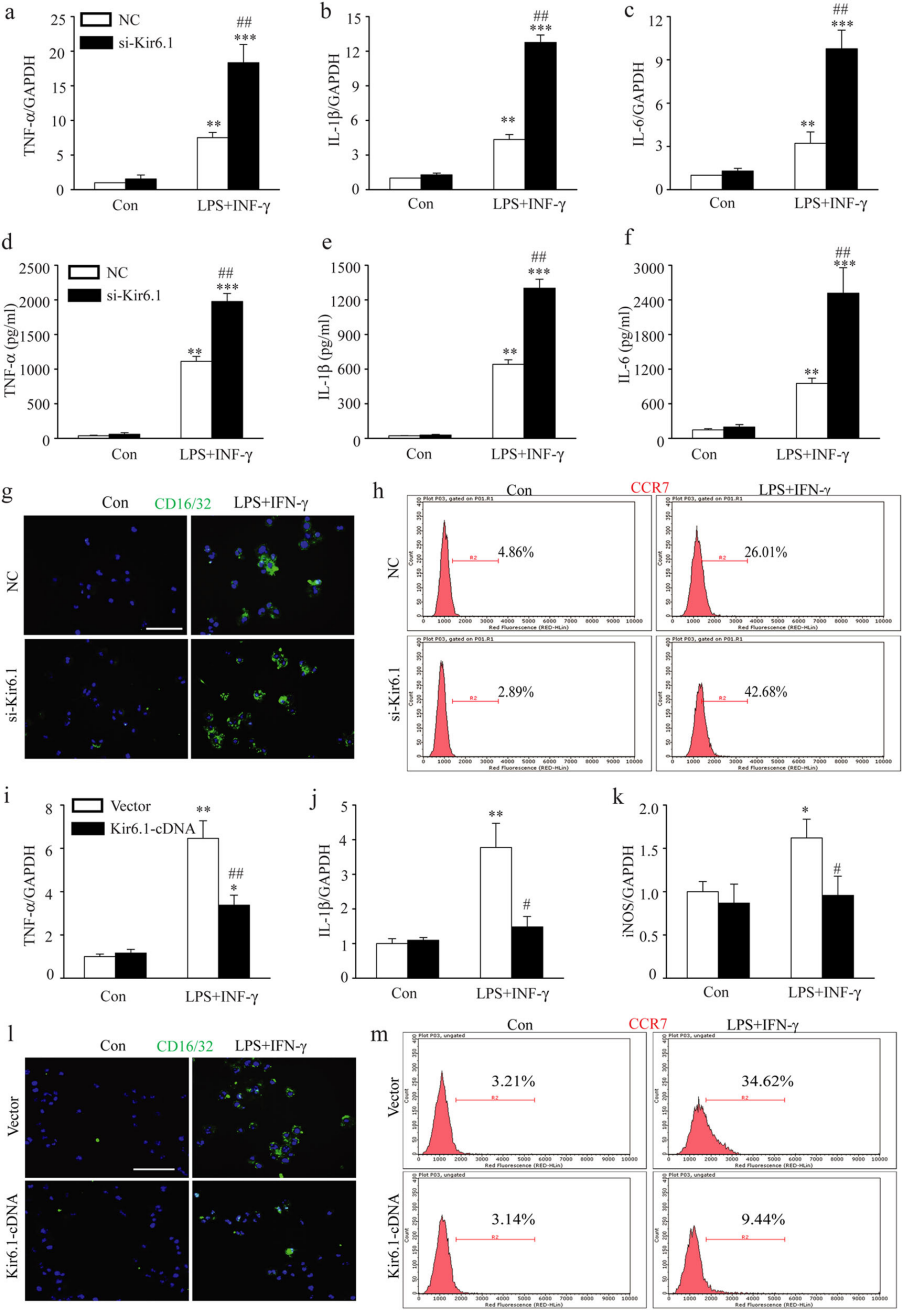
Kir6.1 inhibited M1 microglia polarization. **a**–**h** Kir6.1 knockdown increased the expression of related M1 markers. The expression of TNF-α, IL-1β and IL-6 were assessed by qPCR (**a**–**c**) or ELISA (**d**–**f**) in BV2 microglia. **g** Representative immunofluorescence staining of CD16/32 was visualized under microscopy. **h** The expression of M1 related biomarker CCR7 was detected by flow cytometry. Data are presented as mean ± SEM from four independent experiments, **p < 0.01, ***p < 0.001 versus corresponding control; ^##^*p* < 0.01 versus LPS+INF-γ-treated negative control (NC) groups. **i**–**m** Kir6.1 overexpression inhibited the expression of related M1 markers. The expression of TNF-α (**i**), IL-1β (**j**) and iNOS (**k**) were assessed by qPCR. **l** Representative immunofluorescence staining of CD16/32 was visualized under microscopy. **m** The expression of M1 related biomarker CCR7 was detected by flow cytometry. Data are presented as mean ± SEM from four independent experiments, *p < 0.05, **p < 0.01 versus corresponding control; ^#^*p *< 0.05, ^##^*p* < 0.01 versus LPS+INF-γ-treated vector groups

To further assess the effects of Kir6.1 on M1 polarization, we increased or reduced the expression of Kir6.1 by siRNA-knockdown or transiently expression. The expression of several key pro-inflammatory factors such as IL-1β, TNF-α, and IL-6 were greatly enhanced in Kir6.1 knockdown cells compared to negative control following lipopolysaccharide (LPS) plus IFN-γ challenge (Fig.4a–c). Consistently, ELISA analysis showed a significant higher IL-1β, TNF-α, and IL-6 level in knockdown cells compared with control cells (Fig. 4d–f). Similarly, LPS+IFN-γ treatment significantly increased the expression of M1 markers such as CD16/32 and CCR7 as measured by immunofluorescence and flow cytometry analysis, respectively, but the increase was much more potent in Kir6.1 knockdown cells than that in negative control (Fig. 4g, h). Meanwhile, Kir6.1 overexpression dramatically reduced IL-1β, TNF-α, and iNOS production in response to LPS+IFN-γ stimulation (Fig. 4i–k). Increased Kir6.1 expression also attenuated CD16/32 and CCR7 expression induced by LPS+IFN-γ (Fig. 4l, m). These data indicate that Kir6.1 inhibits M1 microglia polarization.

### Kir6.1 knockdown enhances the activation of p38 MAPK–NF-κB signaling pathway in microglia

**Fig. 5:**
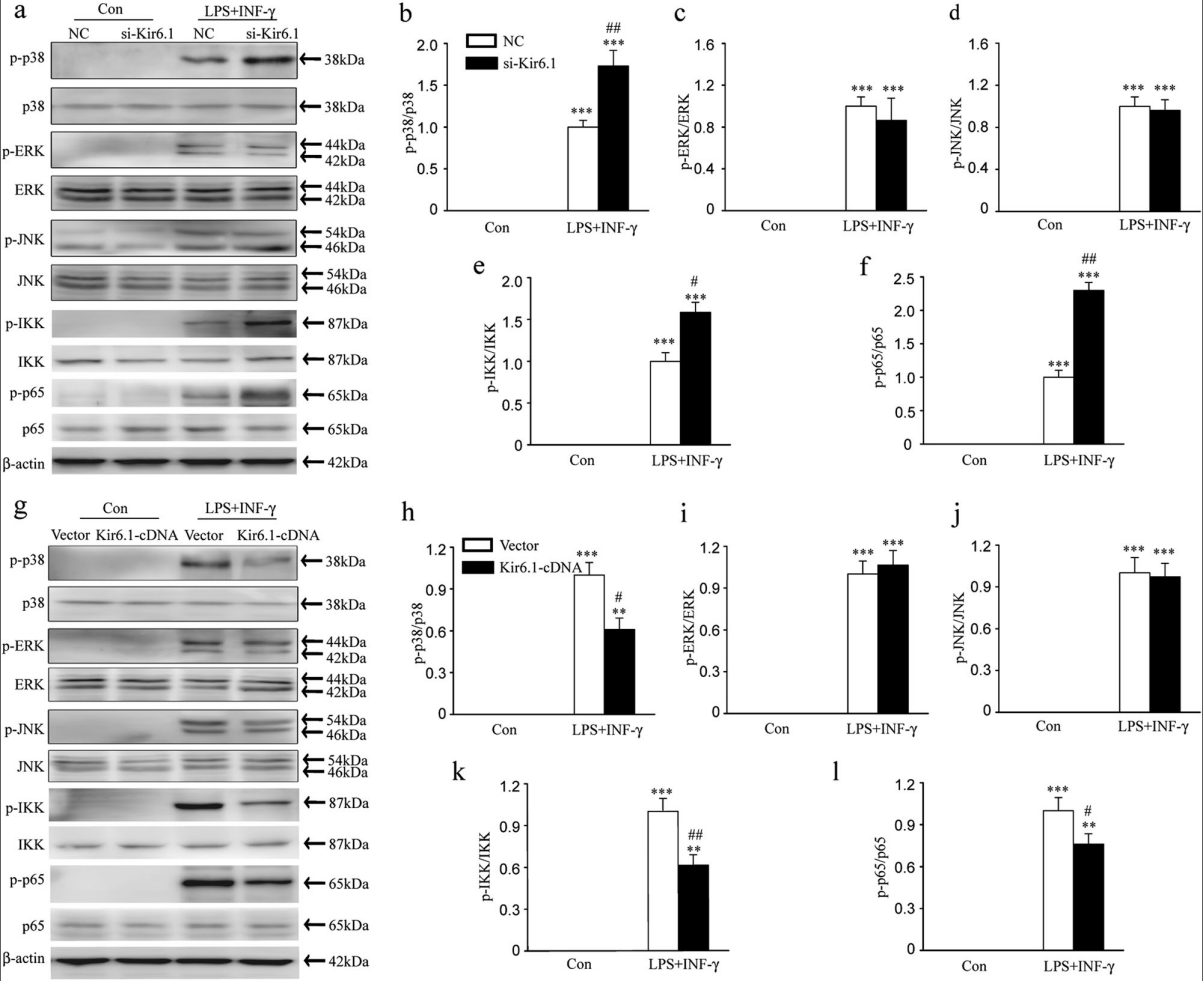
Kir6.1 inhibited the activation of p38 MAPK and NF-κB in microglia. **a**–**f** Kir6.1 knockdown enhanced the phosphorylation of p38, IKK and p65 in microglia treated with LPS+INF-γ. Representative Immunoblot (**a**) and quantitative analysis of the phosphorylation of p38 (**b**), ERK (**c**), JNK (**d**), IKK (**e**) and p65 (**f**) in microglia. Data are presented as mean ± SEM from four independent experiments, ***p < 0.001 versus corresponding control; ^#^*p* < 0.05, ^##^*p* < 0.01 versus LPS+INF-γ-treated negative control (NC) groups. **g**–**l** Kir6.1 overexpression inhibited the phosphorylation of p38, IKK and p65 in microglia treated with LPS+INF-γ. Representative immunoblot (**g**) and quantitative analysis of the phosphorylation of p38 (**h**), ERK (**i**), JNK (**j**), IKK (**k**) and p65 (**l**) in microglia. Data are presented as mean ± SEM from four independent experiments, **p < 0.01, ***p < 0.001 versus corresponding control; ^#^*p* < 0.05, ^##^*p* < 0.01 versus LPS+INF-γ-treated vector groups

To explore the underlying mechanism for Kir6.1/K-ATP channel-regulated microglia phenotype, we examined the expression of MAPK and NF-κB signaling pathway which play crucial roles in M1 microglia inflammatory responses^28^. The phosphorylation of p38, but not ERK and JNK, was significantly enhanced in Kir6.1 knockdown microglia compared to negative control after treatment with LPS+IFN-γ (Fig. 5a–d). Moreover, in response to LPS+IFN-γ stimulation, the phosphorylation levels of IKK and p65 were increased more potently in knockdown microglia than in control cells (Fig. 5e, f). In contrast, increased Kir6.1 expression dramatically inhibited the p38 phosphorylation, but failed to change the phosphorylation of ERK and JNK (Fig. 5g–j). Kir6.1 overexpression also significantly alleviated the phosphorylation of IKK and p65 following the LPS+IFN-γ challenge (Fig. 5k, l). These data demonstrate that Kir6.1 inhibits the activation of p38 MAPK- NF-κB pathway in microglia.

### p38 MAPK mediates M1 microglia polarization and DA neuron degeneration in Kir6.1-deficent mice of LPS PD model

**Fig. 6:**
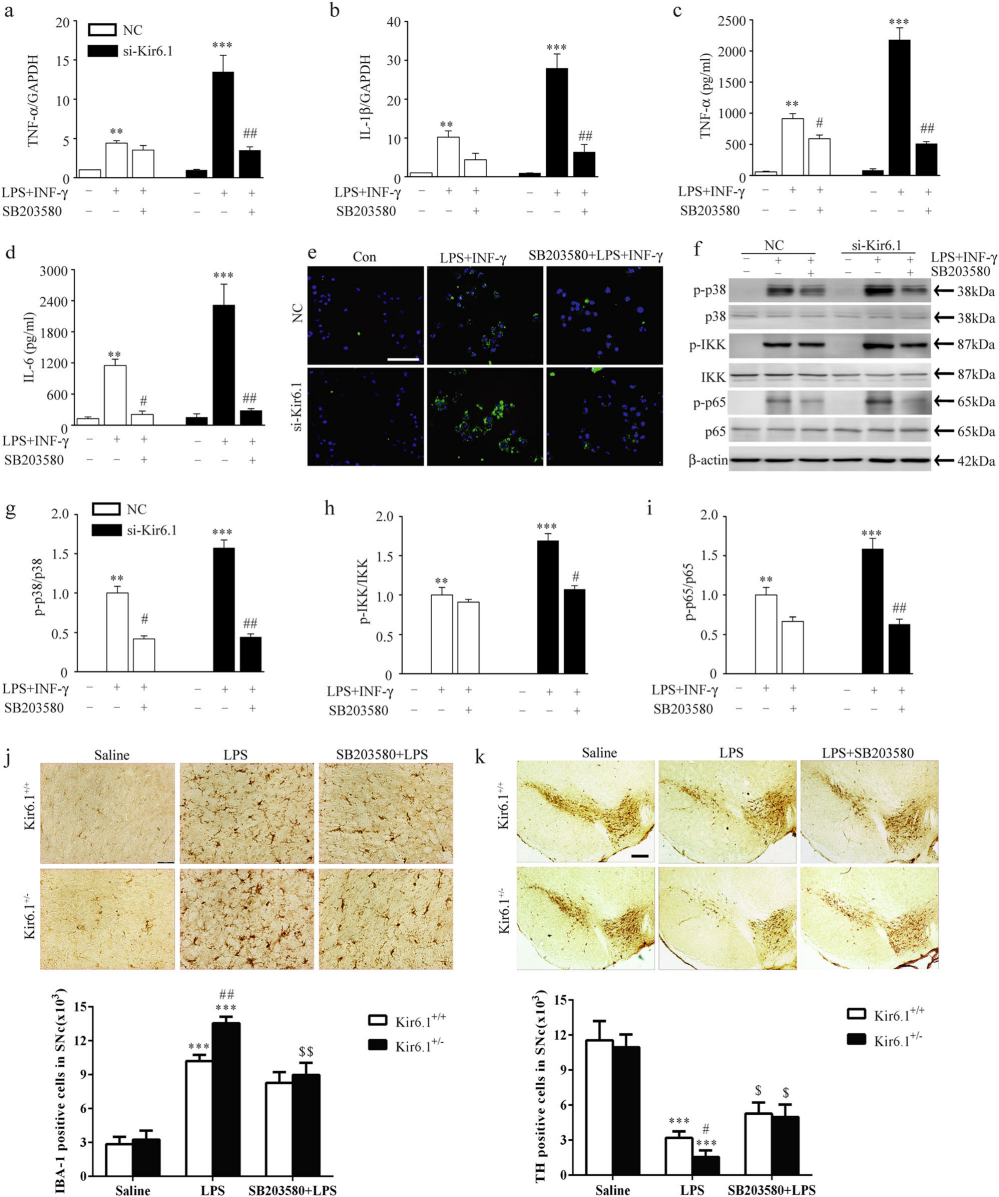
Suppression of p38 MAPK inhibited M1 microglia polarization and alleviated dopaminergic neuron degeneration in Kir6.1-deficent mice of Parkinson’s disease model. **a**–**d** SB203580 reduced the expression of TNF-α (**a**, **c**), IL-1β (**b**) and IL-6 (**d**) were assessed by qPCR or ELISA in Kir6.1- knockdown microglia. **e** SB203580 reduced the expression of CD16/32 in Kir6.1 knockdown microglia. **f**–**i** SB203580 suppressed the phosphorylation of p38, IKK and p65 in Kir6.1 knockdown microglia. Representative immunoblot (**f**) and quantitative analysis of the phosphorylation of p38 (**g**), IKK (**h**) and p65 (**i**) in microglia. Data are presented as mean ± SEM from four independent experiments, **p < 0.01, ***p < 0.001 versus corresponding control; ^#^*p* < 0.05, ^##^*p* < 0.01 versus corresponding LPS+INF-γ groups. **j-k** SB203580 attenuated microglia activation and dopaminergic neuron loss in Kir6.1^+/−^ mice of lipopolysaccharide-induced Parkinson’s disease model. Microphotographs and stereological counts of IBA-1-positive cells (**j**) and TH-positive neurons (**k**) in the substantia nigra compacta. Data are presented as mean ± SEM, ***p < 0.001 versus corresponding control (saline) group; ^#^*p* < 0.05, ^##^*p *< 0.01 versus LPS-treated Kir6.1^+/+^ groups; ^$^*p* < 0.05, ^$$^*p* < 0.01 versus LPS-treated Kir6.1^+/-^ groups. *n* = 4 for each group

Our preceding data have strongly demonstrated that Kir6.1 knockdown promoted M1 microglia polarization and the activation of p38. To further determine whether enhanced p38 activation due to Kir6.1 deletion facilitates M1 microglia polarization, we first treated microglia with p38 specific inhibitor SB203580. SB203580 significantly suppressed the enhanced TNF-α, IL-1β and IL-6 production in Kir6.1-deficient microglia (Fig. 6a–d). SB203580 also reversed the increase in CD16/32 expression due to Kir6.1 knockdown (Fig. 6e). Furthermore, SB203580 dramatically inhibited the enhanced the phosphorylation of IKK and p65 in Kir6.1 knockdown microglia (Fig. 6f–i), suggesting that p38 is upstream of NF-κB. These results indicate that Kir6.1 knockdown promotes M1 microglia polarization via p38 MAPK in vitro.

To determine whether suppression of p38 MAPK could reverse the deleterious effects of ablating Kir6.1 on microglia activation and DA death in vivo, Kir6.1^+/+^ mice and Kir6.1^+/−^ mice were treated with SB203580 before LPS injection. We found that Kir6.1^+/−^ mice displayed a marked increase in the member of microglia overactivation compared with Kir6.1^+/+^ mice after exposure to LPS, which were reversed by SB203580 (Fig.6j). As expected, SB203580 also significantly suppressed the enhanced DA loss in the SNc of Kir6.1^+/-^ mice (Fig. 6k). These results demonstrate that Kir6.1 knockdown enhances the activation of p38 MAPK and promotes M1 microglia polarization, which may contribute to accelerated DA neuron degeneration.

## Discussion

**Fig. 7:**
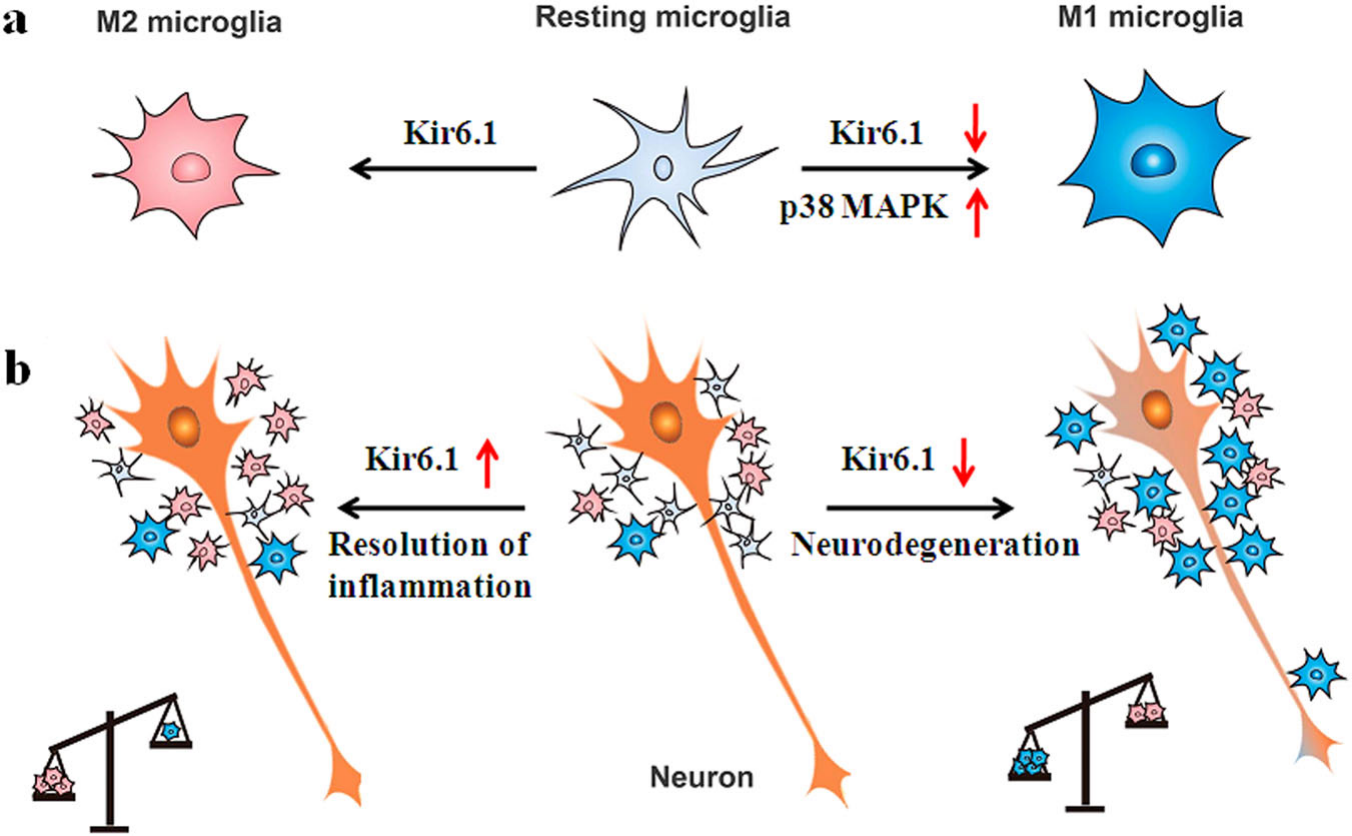
Proposed model of Kir6.1/K-ATP channel involved in regulation of microglia phenotypes and neurodegeneration. **a** Under physiological conditions, microglia exist in a resting state with ramified morphology. They dramatically polarize into M1 or M2 phenotype upon different immunological stimuli or injury. Kir6.1/K-ATP channel switches microglia from the detrimental M1 phenotype toward the beneficial M2 phenotype. Suppression of Kir6.1/K-ATP channel inhibits M2 polarization and exaggerates M1 polarization via activation of p38 MAPK. **b** Suppression of Kir6.1/K-ATP channel compromises neuroprotective effects of M2 microglia and accordingly exaggerates detrimental effects of M1 microglia. The increased ratio of M1/M2 microglia contributes to extensive neuron death and aggravates neurodegeneration

The most important finding presented here is that Kir6.1/K-ATP channel enhances M2 microglia polarization. Kir6.1 knockdown switches microglia from the beneficial M2 phenotype into the detrimental M1 phenotype via p38 MAPK–NF-κB signaling pathway and finally accelerates the DA neuron death (Fig. 7).

Our previous study showed that Kir6.1/K-ATP channel is involved in neurogenesis^29^ and neuroinflammation^25^. In the present study, we found that Kir6.1/K-ATP channel was required and sufficient for M2 microglia polarization. Kir6.1 overexpression was able to increase the gene expression of M2 markers such as Arg1, YM1 and CD206 in response to IL-4 stimulation, suggesting that Kir6.1/K-ATP channel is dispensable for the generation of M2 microglia. Suppression of Kir6.1 inhibited the expression of YM1 and TGF-β in IL-4-treated microglia, whereas enhanced the production of pro-inflammatory cytokines including IL-1β, TNF-α, and IL-6 after treatment with LPS+INF-γ. Moreover, Kir6.1 deletion promoted the production of various pro-inflammatory factors and reduced the anti-inflammatory cytokines such as IL-10 in MPTP-intoxicated mouse of PD models. These results indicate that Kir6.1/K-ATP channel skews M1 toward M2 microglia.

Our study further reveals the underlying molecular mechanism for Kir6.1/K-ATP channel-modulated microglia phenotype. Growing evidence has proposed that MAPK cascade and NF-κB pathway have critical impacts on microglial activation and response^30–32^. In this study, we found that Kir6.1 deficiency enhanced the activation of p38 MAPK and NF-κB but not ERK and JNK in vitro and in vivo. Furthermore, treatment with the p38 inhibitor SB203580 almost abolished the NF-κB activation and exaggerated M1 microglia polarization in Kir6.1 knockdown microglia. More importantly, suppression of p38 could partly reverse the microglia activation and DA neuron loss in Kir6.1^+/−^ mice. These findings suggest that Kir6.1/K-ATP channel inhibits the M1 microglia polarization via suppression of the p38- NF-κB signaling pathway. Thus, it might be of interest to know how Kir6.1/K-ATP channel regulate the activation of p38 MAPK in microglia.

Another important finding described here is that we provide strong evidence further indicating that Kir6.1/K-ATP channel plays an important role in the pathogenesis of PD. Microglia-mediated neuroinflammation is inversely correlated with the DA neuron survival in PD patients^33,34^. In general, activated microglia are prominent and surround DA neurons exhibiting classically activated M1 phenotypes^35^. MPTP is an environmental toxin that causes parkinsonism and is used to establish the animal model of PD^36^. Typical characteristics of M1 phenotype were observed in the MPTP-intoxicated models^37,38^. We first used Kir6.1^+/−^ mice to establish a subacute neurotoxin MPTP-induced PD model and found that Kir6.1^+/^^−^ mice displayed the more DA neuron loss and greater microglial activation in the SNc. Kir6.1^+/−^ mice also showed a significant increase in the ratio of M1/M2 markers. These data indicate that Kir6.1 deficiency accelerates DA neuron degeneration in the MPTP mouse model by facilitating microglia M1/M2 transition. Furthermore, LPS as a classical ligand of toll-like receptors is definitely evoking M1 microglial activation and causes extensive DA neuron death in vivo and in vitro^39–42^. Therefore, an inflammatory LPS-induced PD model was used to further confirm the crucial roles of Kir6.1/K-ATP channel in PD. Consistent with the observed deleterious effects on the DA neuron in MPTP-induced PD model, we found that Kir6.1 deficiency enhanced microglia overactivation and aggravated DA neuron death in SNc of LPS-induced PD model. In summary, our results clearly demonstrate that Kir6.1 deficiency exacerbates DA neuron death in both LPS and MPTP-induced PD models. On the basis of these findings, we propose that Kir6.1/K-ATP channel facilitates the switch of microglia phenotypes from M1 to M2 and ultimately inhibits chronic neurodegeneration in PD. Moreover, it would be interesting to monitor the balance of microglia polarization in the microglia-specific Kir6.1-knockout mice during the generation of PD.

In conclusion, our findings demonstrate that Kir6.1/K-ATP channel switches microglia from the detrimental M1 phenotype toward the beneficial M2 phenotype that may contribute to the immune pathogenesis of PD and that Kir6.1/K-ATP channel may be a promising therapeutic target for PD.

## Materials and Methods

### Mice

Kir6.1^+/−^ mice on a C57/B6 background were made available via the NIH-funded Mouse Mutant Regional Resource Center (MMRRC; http://www.mmrrc.org) for Material Transfer Agreement. Kir6.1^+/^^−^ mice and their littermate wild-type controls (Kir6.1^+/+^) were bred and maintained in the Animal Resource Centre of the Faculty of Medicine, Nanjing Medical University and age-matched adult male mice (4-month-old) used for the experiments. All experiments were carried out in strict accordance with the National Institutes of Health Guide for the Care and Use of Laboratory Animals.

### MPTP-induced PD mouse model

Four-month-old male Kir6.1^+/^^−^ and Kir6.1^+/+^ mice were injected subcutaneously with 20 mg kg^−^^1^ MPTP (Sigma, St. Louis, MO) in saline solution and intraperitoneally with 250 mg kg^−1^ probenecid in DMSO for five consecutive days and left for 3 days^43^. At day 8 post injection the animals were killed. Control mice were treated with saline solution only.

### LPS -induced PD mouse model

Kir6.1^+/−^ and Kir6.1^+/+^ mice were administered intraperitoneal injections of 5 mg kg^−1^ SB203580 (Selleck, S1076, USA) or vehicle (saline) for 7 consecutive days at first LPS injection. The mice were anesthetized with 70 mg kg^−1^ sodium pentobarbital and positioned in a stereotaxic apparatus (Narishige Scientific Instruments, Tokyo, Japan). Using the brain atlas of Paxinos and Watson, LPS (from Escherichia coli, serotype 0111:B4; Sigma, USA) was injected into the SN using a 2-μl Hamilton microsyringe (Switzerland) into the following stereotaxic coordinates: 3.0 mm posterior to bregma, 1.3 mm lateral to the midline, and 4.7 mm ventral to the surface of the dura mater. LPS was dissolved (2.5 mg ml^−1^) in a solution of phosphate-buffered saline (PBS) and injected slowly into one side of the SN in a volume of 2 μl over a period of 5 min, and the needle was kept in place for 5 min after injection^44^. Vehicle (PBS) was injected in a similar manner into the contralateral SN. At day 7 post injection, the animals were killed for the experiments of immunohistochemistry.

### TH and IBA-1 immunostaining

After animals were perfused with 4% paraformaldehyde, brains were dissected out and maintained in 4% paraformaldehyde overnight. They were transferred to 20% sucrose in phosphate-buffered saline (PBS) overnight and then to 30% sucrose overnight till the brain sunk to the bottom of the tube. Serial sections of the brains were cut (30-μm sections) through each entire midbrain using a freezing microtome. Sections were incubated overnight with rabbit mouse monoclonal anti-TH antibody (1:3000; Sigma, St Louis, MO), or mouse anti-IBA-1 antibody (1:1000; Wako, Japan) for the detection of TH and IBA-1, respectively, and then for 1 h with secondary antibodies. Immunoreactivity was visualized by incubation in substrate-chromogen solution (DAB). Control staining was performed without primary antibodies. The total numbers of TH-positive neurons and IBA-1-positive cell numbers in the SNc were obtained stereologically using the Optical Fractionator method with Microbrightfield Stereo-Investigator software (Stereo Investigator software; Microbrightfield).

### Cell culture and treatment

BV2 microglia were purchased from ATCC (American type culture collection), and cultured in Dulbecco’s modified Eagle’s medium (Gibco, USA) supplemented with 10% fetal bovine serum (Gibco, USA) and 1% penicillin/streptomycin. For the induction of M1 phenotype, BV2 microglia were treated with 100 ng mL^−1^ LPS (Sigma, USA) and 20 ng mL^−1^ IFN-γ (Peprotech, USA) for 24 h. For the induction of M2 phenotype, BV2 cells were treated with 20 ng mL^−1^ IL-4 (Peprotech, USA) for 24 h. For pharmacological measurements, the p38 inhibitor SB203580 (10 μM, Tocris, UK) was added to the cell culture medium 1 h before stimulation.

### Cell transfection

BV2 microglia at a confluency of 40–50% in six-well dishes were transfected with the Kir6.1 siRNA oligoribonucleotide sense 5′-GCGACCAAUGUCAGGUCAUTT-3′; antisense 5′-AUGACCUGACAUUGGUCACTT-3′ (Gene Pharma) in OptiMEM (Gibco, USA) using Lipofectamine™ RNAi MAX (Therma fisher, USA) according to the instructions provided. A negative siRNA sequence was used as control. After incubation for 6 h at 37 °C, the transfection mixture was removed and the cells were further incubated in complete growth media for 24 h. BV2 microglia at a confluency of 70–80% in 12-well dishes were transfected with the 1 μg of the full-length mouse Kir6.1 cDNA3.1 plasmid (pcDNA3.1-Kir6.1) or the pcDNA3.1 empty vector in OptiMEM using X-tremeGENE HP DNA Transfection Reagen (Roche, Switzerland) for 6 h. After removal of the media, cells were incubated in complete growth media for 24 h.

### Reverse transcription and quantitative real-time PCR

Total RNA was extracted from cultured cells and SNc of mice with Trizol reagent (Invitrogen, USA). Reverse transcription PCR was carried out using a TAKARA PrimeScript RT reagent kit and real-time PCR was measured using a QuantiTect SYBR Green PCR kit (Qiagen, Germany) with an ABI 7300 Fast Real-Time PCR System (Applied Biosystems, Foster City, CA). GAPDH was used as an internal control for the real-time PCR amplification. The sequences of primers for real-time PCR analysis are as follows: GAPDH forward: CAAAAGGGTCATCTCC; reverse: CCCCAGCATCAAAGGTG. iNOS forward: AATGGCAACATCAGGTCGGCCATCACT; reverse: GCTGTGTGTCACAGAAGTCTCGAACTC. CCL3 forward: ATGCAAGTTCAGCTGCCTGC; reverse: ATGCCGTGGATGAACTGAGG. TNF-α forward: CATCTTCTCAAAATTCGAGTGACAA; reverse: TGGGAGTAGACAAGGTACAACCC. Arg1 forward: GAACACGGCAGTGGCTTTAAC; reverse: TGCTTAGTTCTGTCTGCTTTGC. CD206 forward: GCAGGTGGTTTATGGGATGT; reverse: GGGTTCAGGAGTTGTTGTGG. Ym1 forward: AGAAGGGAGTTTCAAACCTGGT; reverse: GYCYYGCYCAYGYGYGYAAGYGA. IL-1β forward: TCATTGTGGCTGTGGAGAAG; reverse: AGGCCACAGGTATTTTGTCG. IL-6 forward: ATCCAGTTGCCTTCTTCTTGGGACTGA; reverse: TAAGCCTCCGACTTGTGAAGTGGT. TGF-β forward: ACCGCAACAACGCCATCTAT; reverse: GTAACGCCAGGAATTGTTGC.

### ELISA

Cell culture supernatants were assayed for IL-1β, IL-10, TNF-α, IL-6 and TGF-β with ELISA Kits from R&D Systems according to the manufacturer’s instructions.

### Flow cytometry

Cells were incubated with anti-mouse MGL1/2 (CD301a/b) PE-conjugated antibody (R&D, USA), or anti-mouse CD197 (CCR7) PE-conjugated antibody (Ebioscience, USA), and then washed for flow cytometric analyses according to the manufacturer’s instructions (Guava Easy Cyte™ 8, Millipore, USA).

### Immunofluorescence

BV2 microglia were fixed in 4% paraformaldehyde for 15 min. After rinses in PBS, the cells were incubated with the following primary antibody: rat anti-CD16/32 (BD Biosciences Pharmingen, CA, USA) and goat anti-CD206 (R&D Systems, MN, USA) overnight at 4 °C, followed by secondary antibodies for 1 h with extensive washing. Images were captured by Nikon Optical TE2000-S inverted fluorescence microscope.

### Western blotting analysis

Cell lysates were homogenized in lyses buffer (Beyotime, China) and protein concentration was determined by the Bradford assay (Bio-Rad, Hercules, CA, USA). The analysis of protein was performed according to standard SDS–PAGE. Immunoreactive bands were detected by enhanced chemiluminescence (ECL) plus detection reagent (Pierce, Rockford, IL) and analyzed using an Omega 16ic Chemiluminescence Imaging System (Ultra-Lum, CA). The following primary antibodies were used: rabbit anti-Phospho-p38 MAPK (4511, Cell Signaling Technology, USA), rabbit anti-p38 MAPK (8690, Cell Signaling Technology, USA), rabbit anti-Phospho-p44/42 MAPK (4370, Cell Signaling Technology, USA), rabbit anti-p44/42 MAPK (4695, Cell Signaling Technology, USA), rabbit anti-Phospho-SAPK/JNK (4668, Cell Signaling Technology, USA), rabbit anti-SAPK/JNK (9252, Cell Signaling Technology, USA), rabbit anti-Phospho-IKKα/β (2697, Cell Signaling Technology, USA), rabbit anti-IKKβ (8943, Cell Signaling Technology, USA), rabbit anti-Phospho-NF-κB p65 (3033, Cell Signaling Technology, USA), rabbit anti-NF-κB p65 (8242, Cell Signaling Technology, USA).

### Statistical analysis

All data are expressed as means ± SEM. The differences with different treatments and genotypes were determined by one-way or two-way ANOVA, followed by the Tukey’s post hoc test, and were considered as statistically significant at *p* < 0.05.
